# Towards Development of a Standard Terminology of the World Health Organization Classification of Tumors of the Central Nervous System in the Turkish Language, and a Perspective on the Practical Implications of the WHO Classification for Low and Middle Income Countries

**DOI:** 10.5146/tjpath.2022.01584

**Published:** 2022-09-15

**Authors:** Figen Soylemezoglu, Buge Oz, Reyhan Egılmez, Melike Pekmezcı, Suheyla Bozkurt, Ayca Ersen Danyelı, Onder Onguru, Ibrahim Kulac, Tarik Tıhan

**Affiliations:** Hacettepe University, School of Medicine, Ankara, Turkey; Istanbul University, Cerrahpasa School of Medicine, Istanbul, Turkey; Cumhuriyet University, School of Medicine, Sivas, Turkey; Division of Neuropathology, University of California San Francisco, California, USA; Marmara University, School of Medicine, Istanbul, Turkey; Acibadem Mehmet Ali Aydinlar University School of Medicine, Istanbul, Turkey; Anadolu Medical Center, Kocaeli, Turkey; Koç University, School of Medicine, Istanbul, Turkey

**Keywords:** WHO, CNS, Classification, Brain tumors, Gliomas, Low and middle income countries

## Abstract

In our manuscript, we propose a common terminology in the Turkish language for the newly adopted WHO classification of the CNS tumors, also known as the WHO CNS 5th edition. We also comment on the applicability of this new scheme in low and middle income countries, and warn about further deepening disparities between the global north and the global south. This division, augmented by the recent COVID-19 pandemic, threatens our ability to coordinate efforts worldwide and may create significant disparities in the diagnosis and treatment of cancers between the “haves” and the “have nots”.

## INTRODUCTION

‘’*The definitive diagnosis and classification of individual cancers underpins the care of individual cancer patients, as well as research into cancer causation, prevention, diagnosis, and treatment. Traditionally, cancer classification has been based on consensus of histopathological opinion, with very limited consideration of molecular pathology. But new technologies are now transforming the field of pathology more rapidly than at any other time during the past 30 years, and it has become increasingly clear that the traditional approach to cancer classification is insufficient. Our understanding of cancer at a molecular level has now reached the point that this information must be included in diagnoses. Digital pathology and image analysis are also producing new insights, providing quantitative justification of many existing diagnostic criteria while challenging others. The rapid improvement in computer technology, including artificial intelligence, is already producing clinically applicable aids to diagnosis, and this trend is likely to accelerate.*

*There is an urgent need to integrate these facets of diagnosis into cancer classification internationally, and to update the WHO Classification of Tumours on a regular basis. IARC has been responsible for the WHO Classification of Tumours, also known as the WHO Blue Books, since the 3rd edition (2000–2005), which covered all organ sites in 10 volumes. The characteristics of each cancer type, including diagnostic criteria, pathological features, and associated molecular alterations, are described and illustrated in a strictly disease-oriented manner to provide the international standards for diagnosis and cancer research.*’’ (https://whobluebooks.iarc.fr/) (1)

The paragraph quoted above is from the IARC website and frames the necessity for a constant evolution and progress in our efforts to classify neoplasia of different organ systems. Such a classification is “*prerequisite for comparing cancer therapy trials conducted in different centres and countries*” (1), and “*aims to provide a common ground and standard for diagnosis and research of cancers worldwide*” (1). In this perspective, one of the main responsibilities of the WHO classification schemes should be developing “universally applicable” standards that can bridge the communication gap among countries regardless of their economic status or location on the globe.

There is a tremendous amount of information being accrued each day in most scientific disciplines, but in no other area is there more pressure to change and modify practices, standards and guidelines than in medical sciences. There is clearly a limit to how fast changes can be made, and when such changes should be implemented. Studies conducted a decade ago show that our knowledge in medical sciences doubles approximately every 73 days (2), yet validation of this knowledge and modifying everyday medical practice clearly lags far behind this pace. This has also been the case in cancer research and specifically in our study and understanding of the central nervous system (CNS) tumors. WHO tumor classifications attempt to bridge this gap through continuous revisions of existing schemes, and the revised terminology and new nomenclature need to be translated into other languages for everyday practical use across the globe.

In our manuscript, we propose a common terminology in the Turkish language for the newly adopted WHO classification of the CNS tumors, also known as the WHO CNS 5th edition (Table 1). We also comment on the applicability of this new scheme in low and middle income countries (LMIC), and warn about further deepening disparities between the global north and the global south. This division, augmented by the recent COVID-19 pandemic, threatens our ability to coordinate efforts worldwide and may create significant disparities in the diagnosis and treatment of cancers between the “haves” and the “have nots” (3).

**Table 1:** World Health Organization classification of tumours of the central nervous system 5th edition (with Turkish translations).

**Gliomas, Glioneuronal and Neuronal Tumours**	**Gliomlar, Glionöronal ve Nöronal Tümörler**
Adult-type diffuse gliomas	Erişkin-tip difüz gliomlar
Astrocytoma, IDH-mutant	Astrositom,*IDH*mutant
Oligodendroglioma, IDH-mutant and 1p/19q-codeleted	Oligodendrogliom,*IDH*mutant ve 1p/19q kayıplı
Glioblastoma, IDH-wildtype	Glioblastom,*IDH*mutant olmayan
Paediatric-type diffuse low-grade gliomas	Pediyatrik-tip difüz düşük dereceli gliomlar
Diffuse astrocytoma, MYB or MYBL1-altered	Difüz astrositom,*MYB*veya*MYBL1*değişimli
Angiocentric glioma	Anjiosentrik gliom
Polymorphous low-grade neuroepithelial tumour of the young	Genç yaşın polimorf düşük dereceli nöroepitelyal tümörü
Diffuse low-grade glioma, MAPK pathway-altered	MAPK yolağı değişimli düşük dereceli difüz gliom
Paediatric-type diffuse high grade gliomas	Pediyatrik-tip difüz yüksek dereceli gliomlar
Diffuse midline glioma, H3 K27-altered	Difüz orta hat gliomu, H3 K27 değişimli
Diffuse hemispheric glioma, H3 G34-mutant	Difüz hemisferik gliom, H3 G34 mutant
Diffuse paediatric-type high grade glioma, H3 wildtype and IDH wild type	Difüz pediyatrik-tip yüksek dereceli gliom,*H3*ve*IDH*mutant olmayan
Infant-type hemispheric glioma	Infantil-tip hemisferik gliom
Circumscribed astrocytic gliomas	İyi sınırlı astrositik gliomlar
Pilocytic astrocytoma	Pilositik astrositom
High-grade astrocytoma with piloid features	Piloid özellikler gösteren yüksek dereceli astrositom
Pleomorphic xanthoastrocytoma	Pleomorfik ksantoastrositom
Subependymal giant cell astrocytoma	Subependimal dev hücreli astrositom
Chordoid glioma	Kordoid gliom
Astroblastoma, MN1-altered	Astroblastom,*MN1*değişimli
Glioneuronal and neuronal tumours	Glionöronal ve nöronal tümörler
Ganglioglioma	Gangliogliom
Gangliocytoma	Gangliositom
Desmoplastic infantile ganglioglioma / Desmoplastic infantile astrocytoma	Desmoplastik infantil gangliogliom / Desmoplastik infantil astrositom
Dysembryoplastic neuroepithelial tumour	Disembriyoplastik nöroepitelyal tümör
Diffuse glioneuronal tumour with oligodendroglioma-like features and nuclear clusters	Oligodendrogliom benzeri özellikler ve çekirdek kümeleri içeren difüz glionöronal tümör
Papillary glioneuronal tumour	Papiller glionöronal tümör
Rosette forming glioneuronal tumour	Rozet oluşturan glionöronal tümör
Myxoid glioneuronal tumour	Miksoid glionöronal tümör
Diffuse leptomeningeal glioneuronal tumour	Difüz leptomeningeal glionöronal tümör
Multinodular and vacuolating neuronal tumour	Multinodüler ve vakuoler nöronal tümör
Dysplastic cerebellar gangliocytoma (Lhermitte-Duclos disease)	Serebellumun displastik gangliositomu (Lhermitte–Duclos hastalığı)
Central neurocytoma	Santral nörositom
Extraventricular neurocytoma	Ekstraventriküler nörositom
Cerebellar liponeurocytoma	Serebellar liponörositom
Ependymal tumours	Ependimal tümörler
Supratentorial ependymoma	Supratentoryal ependimom
Supratentorial ependymoma YAP1 fusion-positive	Supratentoryal ependimom*YAP1*füzyon-pozitif
Supratentorial ependymoma ZFTA fusion-positive	Supratentoryal ependimom*ZFTA*füzyon-pozitif
Posterior fossa ependymoma	Posterior fossa ependimomu
Posterior fossa ependymoma Group PFA	Posterior fossa ependimomu, PFA grubu
Posterior fossa ependymoma Group PFB	Posterior fossa ependimomu, PFB grubu
Spinal ependymoma	Spinal ependimom
Spinal ependymoma, MYCN-amplified	Spinal ependimom,*MYCN*-amplifikasyonu gösteren
Myxopapillary ependymoma	Miksopapiller ependimom
Subependymoma	Subependimom
**Choroid Plexus Tumours**	**Koroid Pleksus Tümörleri**
Choroid plexus papilloma	Koroid pleksus papillomu
Atypical choroid plexus papilloma	Atipik koroid pleksus papillomu
Choroid plexus carcinoma	Koroid pleksus karsinomu
**Embryonal Tumours**	**Embriyonel Tümörler**
**Medulloblastoma**	**Medulloblastom**
Medulloblastomas, molecularly defined	Medulloblastom, genetik olarak tanımlanan
Medulloblastoma, WNT-activated	Medulloblastom, WNT yolağı baskın
Medulloblastoma, SHH-activated and TP53-wildtype	Medulloblastom, SHH yolağı baskın ve*TP53*mutant
Medulloblastoma, SHH-activated and TP53-mutant	Medulloblastom, SHH yolağı baskın ve*TP53*mutant olmayan
Medulloblastoma, non-WNT/non-SHH	Medulloblastom, WNT ve SHH yolakları dışı
Medulloblastomas, histologically defined	Medulloblastom, histolojik olarak tanımlanan
*Classic medulloblastoma*	*Medulloblastom, klasik*
*Desmoplastic/nodular medulloblastoma*	*Desmoplastik/nodüler medulloblastom*
*Medulloblastoma with extensive nodularity*	*Belirgin nodülarite gösteren medulloblastom*
*Large cell / anaplastic medulloblastoma*	*Büyük hücreli/anaplastik medulloblastom*
**Other CNS Embryonal Tumours**	**Diğer MSS Embriyonel Tümörleri**
Atypical teratoid/rhabdoid tumour	Atipik teratoid/rabdoid tümör
Cribriform neuroepithelial tumour (provisional entity)	Kribriform nöroepitelyal tümör (geçici tip)
Embryonal tumour with multilayered rosettes	Çok katlı rozetli embriyonel tümör
CNS neuroblastoma, FOXR2 activated	MSS nöroblastom, FOXR2 aktive
CNS tumour with BCOR internal tandem duplication	*BCOR*internal ardışık duplikasyon gösteren MSS tümörü
CNS embryonal tumour, NOS	MSS embriyonel tümör, tanımlanmamış
**Pineal Tumours**	**Pineal Bölge Tümörleri**
Pineocytoma	Pineositom
Pineal parenchymal tumour of intermediate differentiation	Orta derecede farklılaşma gösteren pineal parankimal tümör
Pineoblastoma	Pineoblastom
Papillary tumour of the pineal region	Pineal papiller tümör
Desmoplastic myxoid tumour of the pineal region, SMARCB1-mutant	Pineal bölgenin desmoplastik miksoid tümörü, SMARCB1-mutant
**Cranial and Paraspinal Nerve Tumours**	**Kraniyal ve Paraspinal Sinir Tümörleri**
Schwannoma	Schwannom
Neurofibroma	Nörofibrom
Perineurioma	Perinöriom
Hybrid nerve sheath tumours	Hibrid sinir kılıfı tümörleri
Malignant melanotic nerve sheath tumour	Malign melanotik sinir kılıfı tümörü
Malignant peripheral nerve sheath tumour	Malign periferik Sinir kılıfı tümörü
Cauda equina neuroendocrine tumour (CNS paraganglioma)	Kauda ekuina nöroendokrin tümörü (MSS paragangliomu)
**Meningiomas**	**Meningiomlar**
**Mesenchymal, Non-Meningothelial Tumours Involving the CNS**	**MSS’nin Mezenkimal, Non-Meningotelyal Tümörleri**
**Soft Tissue Tumours**	**Yumuşak Doku Tümörleri**
Fibroblastic and myofibroblastic tumours	Fibroblastik ve miyofibroblastik tümörler
Solitary fibrous tumour	Soliter fibröz tümör
Vascular tumours	Vasküler tümörler
Haemangiomas and vascular malformations	Hemangiomlar ve vasküler malformasyonlar
Haemangioblastoma	Hemangioblastom
Skeletal muscle tumours	İskelet kası tümörleri
Rhabdomyosarcoma	Rabdomiyosarkom
Uncertain differentiation	Belirsiz başkalaşım (Gösteren)
Intracranial mesenchymal tumour, FET::CREB fusion-positive	Intrakraniyal mezenkimal tümör,*FET::CREB*füzyon-pozitif
CIC-rearranged sarcoma	*CIC*değişimi gösteren sarkom
Primary intracranial sarcoma, DICER1-mutant	Primer intrakraniyal sarkom,*DICER1*mutant
Ewing sarcoma	Ewing sarkomu
**Chondro-Osseous Tumours**	**Kondro-Osseöz Tümörler**
Chondrogenic tumours	Kondrojenik tümörler
Mesenchymal chondrosarcoma	Mezenkimal kondrosarkom
Chondrosarcoma	Kondrosarkom
**Notochordal Tumours**	**Notokordal Tümörler**
Chordoma	Kordoma
**Melanocytic Tumours**	**Melanositik Tümörler**
Diffuse meningeal melanocytic neoplasms	Difüz meningeal melanositik tümörler
Melanocytosis and melanomatosis	Melanositozis ve melanomatozis
Circumscribed meningeal melanocytic neoplasms	İyi sınırlı meningeal melanositik tümörler
Melanocytoma and melanoma	Melanositom ve melanom
**Haematolymphoid Tumours Involving the CNS**	**MSS’nin Hematolenfoid Tümörleri**
**Lymphomas**	**Lenfomalar**
CNS lymphomas	MSS lenfomaları
Primary diffuse large B-cell lymphoma of the CNS	MSS primer difüz büyük B-hücreli lenfoma
Immunodeficiency-associated CNS lymphomas	İmmün yetmezlik ile beraber görülen SSS lenfomaları
Lymphomatoid granulomatosis	Lenfomatoid granülomatozis
Intravascular large B-cell lymphoma	Intravasküler büyük B-hücreli lenfoma
Miscellaneous rare lymphomas in the CNS	MSS’de bulunan diğer nadir lenfomalar
MALT lymphoma of the dura	Duranın MALT lenfoması
Other low-grade B-cell lymphomas of the CNS	MSS’nin diğer düşük dereceli B-hücreli lenfomaları
Anaplastic large cell lymphoma (ALK+/ALK-)	Anaplastik büyük hücreli lenfoma (ALK+/ALK-)
T-cell and NK/T-cell lymphomas	T-hücreli ve NK/T-hücreli lenfomalar
**Histiocytic Tumours**	**Histiyositik Tümörler**
Erdheim Chester disease	Erdheim Chester Hastalığı
Rosai Dorfman disease	Rosai Dorfman Hastalığı
Juvenile xanthogranuloma	Jüvenil ksantogranülom
Langerhans cell histiocytosis	Langerhans hücreli histiyositoz
Histiocytic sarcoma	Histiyositik sarkom
**Germ Cell Tumours**	**Germ Hücre Tümörleri**
**Tumours of the Sellar Region**	**Sellar Bölge Tümörleri**
Adamantinomatous craniopharyngioma	Adamantinomatöz kraniofarinjiom
Papillary craniopharyngioma	Papiller kraniofarinjiom
Pituicytoma, granular cell tumour of the sellar region and spindle cell oncocytoma	Pituisitom, hipofizin granüler hücreli tümörü ve iğsi hücreli onkositom
Pituitary adenoma /PitNET	Hipofiz adenomu / PitNET
Pituitary blastoma	Hipofiz blastomu
**Metastases to the CNS**	**SSS Metastazları**
Metastases to the brain and spinal cord parenchyma	Beyin ve medulla spinalis parankimine metastatik tümörler
Metastases to the meninges	Meninkslere metastatik tümörler
**Genetic Tumour Syndromes of the Nervous System**	**Sinir Sisteminin Genetik Sendromları**
Neurofibromatosis type 1	Nörofibromatozis tip 1
Neurofibromatosis type 2	Nörofibromatozis tip 2
Schwannomatosis	Schwannomatozis
von Hippel-Lindau disease	von Hippel-Lindau hastalığı
Tuberous sclerosis	Tüberoz skleroz
Li-Fraumeni syndrome	Li-Fraumeni sendromu
Cowden syndrome	Cowden sendromu
Constitutional MMRD syndrome	Yapısal yanlış eşleşme onarım bozukluğu (MMRD) sendromu
Familial adenomatous polyposis syndrome	Familial adenomatöz polipozis sendromu
Naevoid basal cell carcinoma syndrome	Nevoid bazal hücreli karsinom sendromu
Rhabdoid tumour predisposition syndrome	Rabdoid tümör predispozisyon sendromu
Carney complex	Carney kompleksi
DICER1 syndrome	DICER1 sendromu
Familial paraganglioma syndromes	Ailesel paragangliom sendromu
Melanoma-astrocytoma syndrome	Melanom-astrositom sendromu
Familial retinoblastoma	Ailesel retinoblastom
BAP1 syndrome	BAP1 sendromu
Fanconi anemia	Fanconi anemisi
ELP1-medulloblastoma syndrome	ELP1-medulloblastom sendromu

## IS THERE AN IDEAL CLASSIFICATION SCHEME AND WHAT ARE THE FEATURES OF THIS IDEAL CLASSIFICATION?

Biological classifications are incomplete attempts to understand nature, evident in the continuous improvement and revision attempts due to advancing knowledge and understanding. Each rendition of a classification scheme reflects our best efforts to provide a theoretical framework by the “recognized experts of the time”, even though it is often not exactly clear how to select the experts who can assume such a task (4).

How should classifications be made or revised? Are there any criteria or principles that will allow a classification attempt to be more reliable until its next revision? How can classifications be made to be most inclusive so that the pressure for constant revisions or iterations by one group or another is avoided or reduced? These simple questions do not have simple answers (5). In a recent review, it has been suggested that choosing one approach over another fails to recognize that each method serves a different purpose, and that well-defined methods can be ‘‘rolled up’’ into aggregated multidimensional classifications, although the rules and logic about how exactly to undertake this have not been obvious or explicit (5). There is significant divergence in the approaches to biological classifications and what purpose they may serve (5). Therefore, the objectives of any classification initiative could be limited and may not serve all the purposes perceived by the stakeholders (6).

According to Mayr, biological classifications have two major objectives: to serve as the basis of generalizations in all sorts of comparative studies and to serve as the key to an information storage system (6). Mayr also argues whether the achievement of the first objective is reconcilable with the achievement of the second objective, and asks whether the soundest classification for practical use is also the most convenient for information retrieval, i.e. the most comprehensive. When considering pathological classifications, Ackerman & Rosai argued that classification systems need to be “as simple as possible to avoid confusion, and are most valuable when correlated with clinical features, natural history and eventual prognosis” (7,8).

We believe that the major challenge in a tumor classification scheme is the balance between providing the best possible diagnosis by incorporating the latest technologies and the mindfulness of reproducibility, availability, cost, and relevance to current patient care. In order to provide a reliable and valid classification scheme, the endeavor should be at least:

1. Consistent and comprehensive (considering input from all stakeholders),
2. True to real life, i.e. clinically relevant, enabling decisions on treatment,
3. Validated by acceptable scientific methods, coherent and reproducible,
4. Practical, and applicable in all parts of the world, and in diverse settings,
5. Well-accepted, incorporating all stakeholders through participatory efforts including but not limited to meetings with professionals, professional societies, experts of all relevant domains, theoreticians and practitioners; and should achieve an international consensus considering the huge disparities between the global north and the global south.

Even when one considers all the above conditions fulfilled, the validity and reproducibility of each classification system will come under scrutiny over time, and the advances in technology and science coupled with changing conditions and emerging diseases will force modifications (9). In such a background, and with ever-increasing knowledge of molecular mechanisms of neoplasia, the new edition of WHO CNS tumor classification incorporates more than 20 new tumor types and a number of additions and modifications. These modifications appear to follow a strategy outlined in recent publications (10,11). Reportedly, the major challenge for the new revision attempts is “*to meet the acceleration in the acquisition of knowledge and the resulting information overload, while improving the quality of the classification… and to do this faster than ever before, to meet the clinical need for up-to-date diagnosis to benefit patients directly*” (10). We assume that the perspective when meeting such challenges is the entire globe and not the advanced countries where economic and personnel concerns are different from low and middle income countries.

## HISTORICAL PERSPECTIVE AND THE NEW WHO CLASSIFICATION

The initiation of the WHO classification of tumors through a resolution of the WHO executive board in 1956 started an effort to create standard publications to construct a common ground for the diagnosis, treatment and prognostication of tumors worldwide. The first version of the WHO tumors of the CNS edited by Drs. Leslie Sobin and Karl Joachim Zülch was published in 1979, and had a very simple format including a single image per tumor type, accepted terminology and the ICD-O morphology codes (12). The second edition, edited by Paul Kleihues, Peter C Burger and Bernd Scheithauer and titled “Histological typing of tumours of the central nervous system” was published in 1993 (13). This edition had more detail and images for each tumor entity. IARC has taken over the publication of blue books as of the 3rd edition, which was published in 2000 with editors Paul Kleihues and Webster K Cavenee (14). A fourth edition of the CNS tumor classification was published in 2007 with David N Louis, Hiroko Ohgaki, Otmar Wiestler, and Webster K. Cavenee as the new editors (15), and a “revision” of this edition was re-published in 2016 with a total of nine editors (16). The third and fourth editions were more like a textbook with details in histological, clinical features with multiple references and had many co-editors and contributors (17). The reason why the 2016 edition was not a 5th edition, but a “revision” seems to be subjective, and labeling this edition as a “revision” rather than a new edition significantly underestimated the changes that took place between 2007 and 2016. The “revision of the 4th edition was far more than a revision and introduced, for the first time, the concept of “integrated diagnosis”, which began being adopted in everyday surgical practice with some success (18). This revision also included molecular alterations in the definition and diagnostic criteria for certain tumor entities for the first time. While the number of entities defined by genetic alterations and the requirements for advanced and expensive testing were minimal, this revision signaled the incoming avalanche. It was also the first time some parts of the world did not have the technical and financial infrastructure to perform the required molecular analyses, and a rift between the “haves” and “have-nots” has increased (19). Soon after the publication of the 4th edition, a group of experts began writing opinion papers under the title C-IMPACT NOW (the Consortium to Inform Molecular and Practical Approaches to CNS Tumor Taxonomy), trying to inform and further clarify the ambiguities in the 2016 classification (20). The aim was to make the use of WHO 2016 classification easier and more practical, hence the letter “P” in C-IMPACT.

This brought us to the present, when the 5th edition of the CNS blue book was published; first online in December 2021, and in print the following year (21). The current edition of the classification scheme introduces more changes and a greater attempt to standardize and unify the blue books across organ systems. A brief overview of the “new” CNS classification underscores several large and small modifications (21). It appears that there are more than 20 new entities and ~15 revisions in the nomenclature compared to the 2016 edition. A short list of some of these modifications include:

1. For the first time, adult-type and pediatric-type glial tumors have been recognized as different and the glial tumors were divided as “adult-type diffuse gliomas”, “pediatric-type diffuse low grade gliomas” and “pediatric-type diffuse high grade gliomas” and “circumscribed astrocytic gliomas”
2. The term “entity” was replaced by “type” and the term “variant” was replaced by “subtype”.
3. Arabic numerals (1,2,3,4) replaced the Roman numerals (I,II,III,IV) for tumor grades.
4. Each tumor type required listing of “essential” and “desirable” diagnostic criteria (Please find Turkish translation of essential and desirable criteria of some of the most common tumors in Supplementary Table).
5. The mitotic count was no longer reported as per 10 high power magnification fields. Instead, number of mitoses was reported per millimeter square (or 2 millimeter square which roughly corresponds to 10 high power magnification fields).
6. Tumors with different grades, which were listed as separate “entities” were no longer separated as different “types” and given their own chapter. For instance, WHO grade 2 and 3 oligodendrogliomas are now found under the same chapter.
7. The term glioblastoma was restricted to IDH-wildtype diffuse adult gliomas.
8. The term “anaplastic” was dropped from some of the tumor types or subtypes.
9. Glioblastoma diagnosis could be made using molecular criteria (*TERT*promoter mutation,*EGFR*amplification, or gain of chromosome 7 with loss of chromosome10) regardless of histological features if the tumor is considered to be IDH-wildtype diffuse glioma.
10. Many new tumor types are defined (for all new tumor types, please refer toTable 1).
11. Ependymomas were classified based on their location and some molecular features.
12. Several tumor names were changed. The most interesting, and probably impactful change was the introduction of “pituitary neuroendocrine tumor” instead of “pituitary adenoma”, and “cauda equina neuroendocrine tumor” instead of “paraganglioma”.

## REVIEW OF MAJOR TUMOR CATEGORIES

### Adult-Type Diffuse Gliomas

Adult-type diffuse gliomas have been consolidated to three tumor types, which can be further graded histologically (mitotic count, necrosis microvascular proliferation) or by the presence of certain molecular alterations.*Oligodendroglioma, IDH-mutant and 1p/19q-codeleted*is defined by the presence of either*IDH1*or*IDH2*hotspot mutations as well as whole chromosome arm deletions of 1p and 19q (22). Presence of both alterations is required for the diagnosis, and these alterations could be demonstrated using different ancillary tests. These ancillary tests include*IDH1 R132H*-mutation specific immunohistochemistry,*IDH1/IDH2*sequencing (in select cases), FISH, array-CGH or NGS-based analyses for the demonstration of 1p/19q codeletion. Similar to 2016 WHO classification, tumors with elevated mitotic activity or necrosis or microvascular proliferation will be graded as WHO grade 3. In addition, those with*CDKN2A*homozygous deletion will also be designated WHO grade 3 (23–25).

*Astrocytoma*, IDH-*mutant*is defined by the presence of either*IDH1*or*IDH2*hotspot mutation and absence of 1p/19q-codeletion. IDH-mutant astrocytomas often harbor*ATRX*and/or*TP53*mutations (22), and immunohistochemical stains demonstrating loss of nuclear ATRX expression and/or aberrant p53 staining (staining in the majority of tumor nuclei, >50%, or less likely complete absence) can be used as surrogate markers. Since*ATRX*and*TP53*alterations are often seen in a mutually exclusive manner with 1p/19q codeletion, presence of*ATRX*and/or*TP53*alterations can be interpreted as absence of 1p/19q-codeletion in vast majority of the cases (26). However, not all IDH-mutant astrocytomas show ATRX and/or TP53 mutations, and not all mutations are clearly detectable by surrogate immunohistochemical stains; therefore, further molecular testing may be necessary in a limited number of cases. Similar to the 2016 WHO classification, tumors with elevated mitotic activity are graded as WHO grade 3 and those with necrosis or microvascular proliferation are graded as WHO grade 4. In addition, those with*CDKN2A*homozygous deletion are also designated WHO grade 4 (24). However, given the significantly better prognosis associated with the*IDH*mutations, the term “glioblastoma,” is not used for IDH-mutant astrocytomas (24).

*Glioblastoma*, IDH-*wildtype*, WHO grade 4 is defined by the absence of*IDH1/IDH2*mutations and the absence of histone H3 alterations in a diffusely infiltrating astrocytoma which demonstrates one or more of the following histologic or molecular features: microvascular proliferation, necrosis,*EGFR*amplification,*TERT*promoter mutation or entire chromosome gain of 7 with loss of 10 (27,28). It is especially important to confirm the diffuse glioma diagnosis, before employing some of the molecular features for grading such as*TERT*promoter mutations, which are also seen in a wide variety of circumscribed gliomas and glioneuronal tumors. Glioblastomas may demonstrate various histologic patterns, some of which used to be considered variants/subtypes in prior classifications. These include giant cell glioblastoma, small cell glioblastoma, gliosarcoma, glioblastoma with primitive neuronal component, epithelioid glioblastoma among others, each providing a different differential diagnosis that should be considered during diagnostic work-up. Many of these histologic patterns have associations with distinct molecular alterations (i.e.*BRAF*mutations in a subset of epithelioid glioblastomas); however, these associations are not completely specific or sensitive for diagnosis.

Minimum required diagnostic work-up of diffuse glioma varies based on clinical realities including the patient age and imaging characteristics; however, it is strongly recommended to test all diffuse gliomas in adults for*IDH*mutations. This can be limited to immunohistochemical staining, and sequencing could be reserved to a smaller group of patients where clinical and immunohistochemical results are ambiguous. Sequencing for*IDH*,*ATRX*or*TP53*and demonstration of chr 1p/19q codeletion (either by FISH or by array CGH) is strongly recommended as the second step for the differential diagnosis of*IDH*-mutant tumors. These tests can be staggered based on the histologic features and test availability. Since*ATRX/TP53*mutations and 1p/19q codeletion are often mutually exclusive, tumors with one, do not need to be tested for the other. Subsequent assessment of*CDKN2A/B*homozygous deletion for grading purposes is more difficult, given the lack of reliable surrogate marker i.e. p16 staining, and difficulty of determining whether the deletion is hemi- or homozygous on FISH; often necessitating more complex assays (29,30). Whether all*IDH*-mutant astrocytomas and oligodendrogliomas should be tested for*CDKN2A/B*homozygous deletion is another issue that requires balancing the accuracy of grading with resources. It is not practically required if the tumor is already high grade based on the histologic criteria. Some studies suggest that the yield of such testing would be very low in grade 2*IDH*-mutant astrocytomas and therefore, it may be omitted (23,24) while CAP recommendations state “*CDKN2A/B*homozygous deletion testing should be performed on all*IDH*-mutant astrocytomas”(31). Evidence for*CDKN2A/B*testing in oligodendrogliomas is limited, but could be considered in borderline cases, or if there is clinical concern for high-grade tumor on imaging in a case with grade 2 histological features, especially if grading impacts subsequent management.

Any diffuse glioma involving midline structures, regardless of the patient age, should also be tested to rule out H3 K27-altered diffuse midline glioma by immunohistochemistry using H3 K27M mutation-specific antibody along with the H3K27me3 stain (24). H3K27me3 staining maybe more sensitive, given that it will also identify cases with*EZHIP*overexpression without an H3 K27M mutation.

### Pediatric-Type Diffuse Low and High Grade Gliomas

Diffuse gliomas that occur primarily, but not exclusively, in children are termed “pediatric-type diffuse gliomas” and are subdivided into pediatric-type diffuse low-grade gliomas which have a relatively favorable outcome and pediatric-type diffuse high-grade gliomas which typically show an aggressive clinical course. For many of the tumor types, histologic features and the driver molecular alterations need to be combined for a final integrated diagnosis. Regarding pediatric-type diffuse gliomas, some newly recognized entities and some new designations to existing tumor types were added to the classification. Pediatric type diffuse low-grade gliomas include four entities characterized by a diffuse growth pattern.*Angiocentric glioma; diffuse astrocytoma, MYB- or MYBL1-altered; polymorphous low-grade neuroepithelial tumour of the young (PLNTY); diffuse low-grade glioma, MAPK pathway–altered*tumors are listed among pediatric-type diffuse low-grade gliomas. Angiocentric glioma used to be categorized under “other gliomas” in the previous classification. Almost all angiocentric gliomas have a*MYB::QKI*gene fusion and usually show an indolent behavior. Patients with diffuse astrocytoma,*MYB*- or*MYBL1*-altered; present with drug-resistant epileptic seizures. The tumor shows genetic alterations in*MYB*or*MYBL1*and the clinical behavior is benign. PLNTY is a novel entity that is characterized by seizures in young individuals, diffuse growth pattern, oligodendroglioma-like components, calcification, CD34 immunoreactivity, and MAPK pathway alterations. Diffuse low-grade glioma, MAPK pathway–altered, is a poorly defined group of tumors with pathogenic alterations within the MAPK pathway, such as*FGFR1*fusions or*BRAF*mutations without additional molecular alterations.

Pediatric-type diffuse high-grade gliomas comprise four tumor types that include diffuse midline glioma, H3 K27-altered;*diffuse hemispheric glioma, H3 G34-mutant; diffuse pediatric-type high-grade glioma, H3-wildtype and IDH-wildtype*, and*infant-type hemispheric gliom*a. The term “glioblastoma” is no longer used for pediatric-type high-grade diffuse gliomas. The term used in the 2016 scheme, ‘’diffuse midline glioma, H3 K27M-mutant’’, has been changed to*diffuse midline glioma H3-K27 altered*, reflecting the recognition that other molecular alterations such as*EZHIP*overexpression may also lead to H3 K27M-like changes. Diffuse pediatric-type high-grade glioma, H3-wildtype and IDH-wildtype tumors show high-grade histologic features and do not involve H3 and IDH alterations. Infant-type hemispheric glioma is a novel entity that occurs in newborns and infants. These tumors have fusions of*ALK, ROS1, NTRK1/2/3,*or*MET*genes. Some of these tumors may have been classified as desmoplastic infantile astrocytoma or ganglioglioma in the past, leading to different interpretations of the prognostic characteristics of these low-grade tumors.

### Circumscribed Astrocytic Gliomas

WHO CNS 2021 combines all expansile/non-diffuse astrocytic tumors under the title circumscribed astrocytic tumors that includes the tumors that were previously categorized as “other astrocytic tumors” and “other gliomas”. This group includes*pilocytic astrocytoma (PA), pleomorphic xantoastrocytoma (PXA), subependymal giant cell astrocytoma (SEGA), chordoid glioma (CG), and astroblastoma MN1-altered*. In addition, this group includes a new tumor type called*high-grade astrocytoma with piloid features*. The main reason for using the term “astrocytic glioma” as opposed to astrocytoma stems from the fact that two tumor types in this group, astroblastoma and chordoid glioma, appear to have more ependymal features in addition to astrocytic qualities.

PAs are still defined on histological grounds, and commonly characterized with an internal duplication in the BRAF gene that also causes a fusion between*BRAF*and*KIAA1549*. The only accepted subtype within PA is the “Pilomyxoid Astrocytoma” that has not been assigned a grade due to limited number of comprehensive studies. Similarly, “pilocytic astrocytoma with anaplastic features” is mentioned but is not assigned as a subtype or given a grade due to lack of comprehensive data. These modifications are left for the next iteration of the WHO classification. PXAs are recognized by*BRAF*p.V600E mutations that accompany homozygous*CDKN2A/2B*deletion. Astroblastoma is characterized by MN1 gene fusions and no grade is assigned for this tumor due to the lack of comprehensive data (32). Chordoid glioma characteristically harbors*PRKCA*mutations, specifically p.D463H mutations (33).

One of the most controversial additions to the WHO CNS 2021 is high-grade astrocytoma with piloid features. There are too few reports on this tumor type, and according to the WHO this tumor can only be diagnosed by methylation profiling since it does not have well-defined clinical, radiological, histological or genomic features (34). This tumor is not assigned a grade, and unlike most other tumors in this group it is associated with aggressive behavior. The same group of authors who reported the single publication on high-grade astrocytoma with piloid features also suggested that these tumors may have significant overlap with the so-called cerebellar glioblastomas (35). This tumor appears to be more of a methylation cluster than a true tumor entity and it may undergo significant modification before the next WHO iteration.

Circumscribed astrocytic gliomas can often be readily recognized with the help of clinical, radiological, histological and immunohistochemical features. The exceptions are astroblastomas and chordoid gliomas, which may require genomic characterization to identify*MN1*or*PRKCA*mutations, respectively.

### Glioneuronal and Neuronal Tumors

Tumors with a neuronal component have been grouped together under “Neuronal and Glioneuronal Tumors” in the 5th edition with an addition of three new types, of which one is provisional. One of the new tumor types is the myxoid glioneuronal tumor, characterized by proliferation of oligodendrocyte-like cells embedded in a prominent myxoid stroma. The tumors are typically located in the septum pellucidum involving the lateral ventricle. Multinodular and vacuolating neuronal tumor was listed under gangliocytoma in the 2016 classification, and is a benign tumor consisting of discrete and coalescent nodules within the deep cortical ribbon and superficial subcortex of the temporal lobes associated with seizures. Those nodules are composed of monotonous neuronal elements characteristically showing vacuolar changes. Diffuse glioneuronal tumour with oligodendroglioma-like features and nuclear clusters (DGONC) is a provisional tumor type with an ambiguous morphology for which methylation profiling is required. Paraganglioma, which had been discussed under Neuronal and Glioneuronal tumors in the previous editions, has been renamed as cauda equina neuroendocrine tumor and moved to the ‘’Cranial and Paraspinal Nerve Tumors’’ section in the new edition. It is noteworthy that for most of the tumors listed under neuronal and glioneuronal tumors, diagnosis can be made by careful morphological assessment along with judicious use of immunohistochemistry. Neuronal markers (synaptophysin, NeuN, chromogranin, Hu, non-phosphorylated NFP, internexin A), glial markers (GFAP, S100, OLIG2), CD34, p16, and BRAF VE1 can be used in the diagnosis, considering the diagnostic expression profiles in the literature. Molecular workup has been advised only for the exceptional unresolved cases in this category of tumors. PRKCA gene fusion for papillary glioneuronal tumor, and the FGFR1::TACC1 fusion for extraventricular neurocytoma, as well as the KIAA1549::BRAF fusion and chr 1p deletion for diffuse leptomeningeal glioneuronal tumor have been listed as the essential criteria. With the exception of these three neurocytic neoplasms, most of the glioneuronal tumors are low-grade epilepsy associated tumors with characteristic clinical, radiological, and histological features as well as immunohistochemical profiles, and surgical treatment that results in seizure control is considered curative.

### Ependymal Tumors

Ependymal tumors are classified based on the combination of histopathological and molecular findings and the anatomical site, and include the supratentorial, posterior fossa, and spinal ependymoma groups. Supratentorial ependymomas also include two tumor types that harbor*ZFTA*(*C11orf95*, previously known as REL-A fusion tumors) or YAP1 gene fusions. Posterior fossa ependymomas include the posterior fossa group A (PFA) and posterior fossa group B (PFB) tumors. PFA and PFB are typically distinguished by their global levels of H3 p.K28me3 (K27me3), but to a large extent PFA corresponds to pediatric and PFB corresponds to adult posterior fossa ependymomas. Some spinal ependymomas are defined by*MYCN*amplification, which portends a poor prognosis. While the most common genetic alterations in spinal ependymomas are damaging*NF2*mutations, some spinal ependymomas do not harbor single nucleotide variants or fusions that could be used for “molecular” diagnosis. Papillary, clear cell, and tanycytic ependymomas, which were histological subtypes in the previous classification, are listed as distinctive patterns in the histopathological description of ependymomas. For molecular diagnosis, it is essential to determine the molecular alterations required for each molecular group besides the morphological and immunohistochemical features compatible with ependymoma. Z*FTA (C11orf95)*or*YAP1*gene fusions and*MYCN*amplification can be demonstrated by FISH or sequencing methods, while the decision for PFA/PFB groups can be simply made with the use of H3K27me3 antibody and patient age. For practical purposes, we do not recommend using methylation profiling for posterior fossa ependymomas except for the rare case in which the ependymal nature of the tumor cannot be established. While methylation profiling has been included into the essential criteria for the diagnosis of this group, this may be necessary only on rare occasions. If methylation profiling is regarded as obligatory, it is likely that the diagnosis of PFB group ependymomas will be problematic in LMICs.

Similar to the previous classification, ependymal tumors are graded as grade 2 or 3 according to their histopathological features, but the word “anaplastic” has been removed from the terminology. However, since the term anaplastic is used occasionally in the WHO 2021 classification, and the term is engrained in the practice of neurooncology, we do not see any harm in including the term “anaplastic ependymoma” in the final diagnosis.

Myxopapillary ependymoma (MPE) and subependymoma have been retained as histopathologically defined tumor types. MPEs, which were classified as grade I in the previous classification, are classified as grade 2 in 2021, but data for this rationale still seem to be limited. This partly molecular classification is likely to engender confusion until sufficient data on the prognosis are available from prospective clinical trials (36).

### Embryonal Tumors

For medulloblastomas that constitute the majority of embryonal tumors in the CNS, the WHO 5th edition has remained similar to the CNS WHO revised-4th edition (16) and the Haarlem consensus report (37). There are two different categorizations of medulloblastomas based on molecular or histological features. A nonspecific designation, “medulloblastoma, NOS” is saved for instances where further histological or molecular characterization cannot be made (see Table I).

Recent studies based on methylation and transcriptomic profiling have suggested numerous subtypes for medulloblastoma, but these have not been included in the WHO 5th edition due to incomplete and sometimes conflicting findings. The current consensus includes 4 well-recognized molecular types; WNT-activated-Medulloblastoma, SHH-activated-Medulloblastoma with and without TP53 mutation, and the others classified under the “non-WNT/non-SHH group. While other subgroupings exist in the literature, there are limited data to incorporate any of these attempts to further subcategorize medulloblastomas into the current classification (38). It is currently not clear whether further subclassification based on methylome and transcriptome data would provide any benefit to the existing approaches in the management of medulloblastomas (39).

Practically, differentiation of WNT-activated, as well as TP53 wild-type and TP53 mutant SHH-activated types may be important, and immunohistochemical stains including B-catenin, GAB1, YAP1, ALK, LEF1, and p53 can help segregate an overwhelming majority of medulloblastomas in everyday pathology practice. TP53 mutant SHH-activated medulloblastoma reportedly has a less favorable prognosis than with wild-type TP53.

The new edition continues to recognize the predictive value of histological groupings, often reported to have significant correlation with molecular groups of medulloblastomas (Table 1). For example, desmoplastic/nodular medulloblastomas as well as those with extensive nodularity are almost always included in the SHH-activated Group. In addition, WNT-activated medulloblastomas often have classical morphology, and the large cell/anaplastic medulloblastomas are included in either the SHH-activated group or non-WNT/nonSHH group (38).

There are additional modifications in the other CNS embryonal tumors category that were not present in the 4th edition. A few new tumor types were added and some previous categories were excluded. The new tumor types, partly based on their molecular/genetic features include CNS neuroblastoma,*FOXR2 activated*(40),*CNS tumor with BCOR internal tandem duplication*(41) and the provisional tumor type*cribriform neuroepithelial tumor (CRINET)*(42) that was included in the classification scheme with only a single publication. FOXR2-activated neuroblastomas seem to correspond to the CNS neuroblastomas and ganglioneuroblastomas present in earlier classification schemes. CNS tumor with BCOR internal tandem duplication is a novel tumor type that will require further characterization and its origin. Occasionally, immunohistochemical staining with the BCOR antibody can be useful. There are very limited data on the tumors classified as CRINET, and loss of SMARCB1 (INI1/BAF47) has been reported as characteristic of this tumor type. It remains to be seen whether such tumors constitute a distinct entity (i.e. tumor type) or should be classified elsewhere as a subtype.

Tumors already in the 4th edition,*atypical teratoid/rhabdoid tumor*(AT/RT) and*embryonal tumor with multilayered rosettes*(ETMR) have seen minimal modifications in the new classification. The diagnosis of AT/RT requires demonstration of*SMARCB1*or*SMARCA4*mutation, even though it is possible to render this diagnosis using immunohistochemical results in the right clinical setting. In the developing world, incorporating patients’ clinical and radiological examination results along with BAF47 (SMARCB1) and DRG (SMARCA4) staining should be sufficient. ETMR can be diagnosed often in the right histological setting and positivity with the LIN28A antibody, even if the C19MC anomaly could not be shown genetically. Especially in the face of treatment protocols available even in low resource settings, it may not be wise to leave the diagnosis as “Embryonal Tumor, NOS” which could mean any other embryonal tumor including medulloblastoma. Again for LMIC, we do not see the absolute necessity for demonstrating the C19MC or DICER1 mutations in the right clinical, radiological and immunohistochemical setting, and these analyses could be used more judiciously in difficult cases.

### Meningiomas and Mesenchymal Tumors

According to the 5th edition, all meningiomas are classified under a single type tumor type with 15 morphological subtypes. Atypical or anaplastic (grade 2 and 3) meningioma criteria are defined without regard to the subtypes. As in previous editions, chordoid and clear cell meningioma are classified as WHO grade 2, while rhabdoid and papillary morphology are not automatically considered within the anaplastic category. The tumor grading was based on overall morphological features with a few exceptions. WHO identifies numerous molecular markers associated with specific subtypes, such as loss of nuclear SMARCE1 expression in clear cell meningiomas,*BAP1*mutations and loss of BAP1 staining in rhabdoid and papillary subtypes,*KLF4/TRAF7*alterations in secretory meningiomas, as well as*TERT*promoter mutations or*CDKN2A/B*homozygous deletions in anaplastic tumors. Most of these alterations need to be analyzed via sequencing, and some including*CDKN2A/B*losses could be investigated by fluorescence in-situ hybridization (FISH). Another poor prognostic group has been associated with loss of H3K27 trimethylation, as demonstrated with H3K27me3 antibody, but data on this issue are still preliminary.

The grading of meningiomas has not changed much from the 2016 scheme, with the exception of not considering papillary or rhabdoid meningioma automatically as WHO Grade 3 tumors. In addition, there are now molecular criteria for the designation of anaplastic (i.e. grade 3) meningiomas. The criteria for anaplastic meningioma now include*TERT*promoter mutation as well as*CDKN2A/B*homozygous deletion. While the WHO recommends that tumors with*TERT*promoter mutation and*CDKN2A/B*homozygous deletion be designated grade 3 neoplasms, there is significant cost associated with these analyses and the practical impact of this on clinical care has not been determined. It will be critical to further define how this would alter prognostication or patient management, and the level of improvement in patient outcomes should define the necessity of these analyses. Such studies have not been conducted to date and are under way.

Mesenchymal, Non-Meningothelial Tumors now include*hemangioblastomas*and*chordomas*in addition to the classical soft tissue and bone neoplasms. The diagnostic approach to these neoplasms are the same as reported in the WHO Classification of Tumours of Soft Tissue and Bone (43). There are three new tumor types:*Intracranial mesenchymal tumor, FET-CREB fusion-positive*(provisional) (44), CIC-rearranged sarcoma (45), and*primary intracranial sarcoma, DICER1-mutant*(46). All of these new entities are poorly defined, and have limited clinical characterization and no specific treatment. It is again not clear whether the recognition of these tumors beyond “high-grade sarcoma” has any practical clinical significance (44–46). One significant distinction from the WHO Classification of Tumours of Soft Tissue and Bone is the grading and characterization of solitary fibrous tumors (previously also referred as hemangiopericytoma). The revised grading scheme includes the mitotic rate (greater than 2.5 mitoses per mm2 or 5 per 10 high power magnification fields) and the presence of necrosis while the Bone and Soft Tissue scheme uses a “multivariate” model (47).

### Genetic Tumor Syndromes

A total of 19 genetic syndromes are listed in the new edition of the WHO classification of CNS tumors. This list includes 8 new additions to the existing group of syndromes from 2016, including*Carney complex, DICER1 syndrome, familial paraganglioma syndrome, melanoma-astrocytoma syndrome, familial retinoblastoma, BAP1 tumor predisposition syndrome, Fanconi anemia, and ELP1-medulloblastoma syndrome*. Unlike the previous edition, Turcot syndrome was not included as a tumor predisposition syndrome and its use as a term was not recommended. Brain tumor polyposis syndrome type 1/mismatch repair cancer syndrome has been replaced by constitutional mismatch repair deficiency syndrome (CMMRD) defined by biallelic germline mutations in one of four mismatch repair genes (MLH1, PMS2, MSH2, and MSH6).*Familial adenomatous polyposis*1 (FAP1) syndrome has been defined as an autosomal dominant cancer syndrome caused by an inactivating germline mutation in the*APC*gene. A subset of these patients that develop primary brain tumors (principally medulloblastoma with WNT activation) are currently referred to as having brain tumor polyposis syndrome 2 (BTP2).

The Carney complex, DICER1 syndrome, familial paraganglioma syndromes, BAP1 tumor predisposition syndrome, and familial retinoblastoma have also been covered in other WHO classification schemes for endocrine, skin, and eye tumors.

Familial paraganglioma syndromes are a group of inherited cancer syndromes characterized by the presence of paragangliomas (including pheochromocytoma), and the loss of SDHB immunoreactivity has a high predictive value for SDHB, SDHC, or SDHD mutations. BAP1 tumor predisposition syndrome is caused by pathogenic germline variants in the BAP1 tumor suppressor gene and characterized by a predisposition to various tumors including meningioma. Many BAP1-mutant meningiomas have overt rhabdoid cytomorphology, but the histology can be diverse, including epithelioid-type cells and papillary growth. Loss of*BAP1*immunoreactivity in tumor cell nuclei readily identifies mutations with reasonable accuracy. Concordance between immunohistochemistry and genotyping is high but incomplete. Familial retinoblastoma, which has been well-known for decades, is caused by germline*RB1*pathogenic variants often presenting with bilateral (sometimes trilateral) retinoblastoma also covered as a title in the new edition. Melanoma-astrocytoma syndrome is caused by germline pathogenic variants of the CDKN2A tumor suppressor gene (heterozygous) and characterized by an increased risk of multiple neoplasms (cutaneous melanoma, pancreatic cancer, and squamous cell carcinoma of the oropharynx) including astrocytomas and nerve sheath tumors. Fanconi anemia is a clinically and genetically heterogeneous disorder where the predominant CNS tumor manifestation is medulloblastoma, resulting from biallelic pathogenic germline variants in either*BRCA2*or*PALB2*. The ELP1-medulloblastoma syndrome is caused by heterozygous pathogenic germline variants in the ELP1 gene and characterized by an increased risk of sonic hedgehog (SHH)-activated medulloblastoma during childhood. Absence of the ELP1 gene and protein expression in resected tumor material allows for the identification of patients with the ELP1-medulloblastoma syndrome.

## CHALLENGES ARISING WITH THE NEW CLASSIFICATION

The substantial modifications in the new classification raise a number of concerns for the practical utility of the 5th edition. First and foremost, what started as a histological or histopathological classification, by choice, moved away significantly from histological data and evidence. While molecular findings have been of major significant advances in recent years, recognition of many such molecular alterations have relied on the accurate histological interpretation as well as experience and expertise in this area. It would have been desirable not to reduce the histological information to short paragraphs and allow better recognition of the histological spectrum of each tumor type, which are often helpful for the practicing pathologists in low resource settings (20).

The adoption of the “integrated diagnosis” with sophisticated molecular analyses as components of the “essential criteria” is a distinct diversion from most WHO classification systems for which some tumor types are considered as unique regardless of their molecular features, and histological factors are clearly important in their diagnosis and prognostication (48–50). If we are to move away from a primarily histological classification, then maybe true integration could be achieved with significant participation of neurosurgeons, neurooncologists and neuroradiologists, rather than token representations, but this does not appear to be a major concern for the current version. While integrated diagnosis has successfully merged molecular information into decision making, clinical, radiological and histological components that could be considered critical to a truly integrated diagnosis have been left to brief descriptive paragraphs and has significantly diminished in quality and quantity compared to earlier editions (15,16).

The choice of the term “tumor type” instead of “entity” and incorporating multiple grades of tumors into a single tumor type is quite interesting, as are the definitions for “essential criteria” and “desirable criteria”. While in most other blue books, low grade and high grade examples of tumors, such as low/high grade chondrosarcomas, are listed in their own respective chapters and considered different ‘types”, grade 2 IDH-mutant astrocytomas (low grade) and grade 4 IDH-mutant astrocytomas (high grade) are listed under the same tumor type. While the general goal is to standardize approaches across blue books, this seems to function in an opposite direction (51). This approach also blends the features of high grade and low-grade tumors in the same paragraph, making it confusing to suggest whether all such features (e.g. radiological or clinical information) are relevant to all grades of the entity. On the other hand, it would have been desirable if the essential and desirable criteria were selected using some scientific methodology rather than leaving it to the authors’ and ultimately, the editors’ choice.

Some suggest that the concept of disease entity is theoretical, not clearly definable by pure observation, and has to fulfill the principles of completeness and unambiguousness (52). This implies that every single case is an instance of one disease entity and is subsumed by the one single entity, and if the disease entity or tumor type is to be the central concept in classifications, then each entity demands rigorous review and validation (5,9). A recent review has suggested “some” principles for the definition of an entity that included “*a)* *significant number of cases describing the entity*” suggesting the necessity to define what is implied by the word “*significant*”; “*b) adequate number of independent studies reporting the entity” not specifying what would be deemed “adequate*”; “c) practical utility of the proposed entity because of its clinical relevance or uniqueness”, again, being vague on the concept of “practical utility”; “d) unique biological background…mutation, transcriptomic signature or specific immunohistochemical profile”; and finally “*e) in the future, artificial intelligence approaches…may lead to a unique definition of the entity in question*” again not being clear as to whether artificial intelligence approaches in question are easily definable or acceptable set of methods (11). This definition leaves a lot to the subjective judgment of individuals as to how an entity is decided to have fulfilled “some” of these criteria (11).

There are more than 20 new entities, i.e. new tumor types in the 5th edition, and their identification as new types seems to follow different strategies. Historically, there has been rigorous debate and validation, and the presence of distinctive clinical and pathological information was obligatory to consider a tumor as a new disease “entity” (53,54). In addition, new entities were characterized by their histomorphological spectrum, clinical characteristics, demographic features, and biologic behavior prior to 2016, and with all those and (some) molecular criteria by 2016. Earlier versions may have been devoid of significant molecular information, yet the entity inclusion criteria were meticulous and were based on reproducibility and validation studies, i.e., two or more reports from different/independent institutions were considered mandatory. We are not certain this is the case for some of the new entities included in the 5th edition.

One additional issue is a serious concern in the application of criteria as to what constitutes a new “entity” or “tumor-type” and that is the quality and quantity of publications that are acceptable when making this decision (6,9). For instance, if we were to accept simply the number of publications on a subject as sufficient to adopt a particular idea within classification systems, then the recent publications using “convolutional neural networks” (55,56) or “fine-tuned GoogLENet” approaches (57) should allow us to propose alternate and equally effective classifications based on radiological evidence alone. In a brief review of Pubmed publications, one can find more than a dozen studies published in 2021 alone, suggesting that AI based algorithms could replace conventional or molecular schemes in brain tumor classification. Such a proposition highlights that even when some ideas are accepted in respectable journals, their application or acceptance in practice requires more than simply being published.

Another challenging issue is to variably subtracting location names or the anaplastic designation in certain entities (e.g. rosette-forming glioneuronal tumor of the fourth ventricle and anaplastic oligodendroglioma), but retaining and/or incorporating location information and anaplastic designation in others (cauda equina neuroendocrine tumor or anaplastic meningioma) (21). Thankfully, most such modifications do not significantly impact patient care even though they may significantly alter the results of epidemiological studies. Coding strategies for tumors with morphology, location and procedure in systems such as SNOMED may need to be re-evaluated for consistency with the new 5th edition. Historically, there has been discrepancy between WHO classifications of CNS tumors and other systems such as ICD and SNOMED, and a solution is yet to be found (58).

It has been easy to recognize the reliance of publications of the C-IMPACT group by the WHO working groups and the final text of the WHO CNS 5th edition (21). C-IMPACT publications provide valuable opinions, but present no original data and only partially attempt to demonstrate the validity (but not reproducibility) of the assertions made. For example, the utilization of the terms not otherwise specified (NOS) and not elsewhere classified (NEC) was discussed in the first C-IMPACT publication with only partial clarification as to how one should choose one term over the other (59). There was also limited corroborating evidence and no second publication or independent study to substantiate the validity and assess the reproducibility of these assertions until their adoption by the WHO. In addition, it is not clear to the authors what constitutes a “full molecular work-up” (59) and when a molecular work-up is considered “full”. Furthermore, whether this will change any clinical practice trends or improve or hamper clinical care is not clear. It is however clear, that the NOS diagnoses used in LMIC for lack of resources create significant problems to pathologists and much dissatisfaction among neurooncologists (personal correspondence). We have begun to quantify and determine the degree of challenges resulting from the use of these terms within the neurooncology community in our country, and hope that others may also attempt to answer this question.

Other publications of C-IMPACT, even though they were mentioned simply as expert opinions to provide more practical use of the WHO 2016 classification, have been adopted in the current classification scheme with limited corroborating data or publications from other groups validating these assertions, at least prior to adoption by the WHO (24,26,28,36,60,61).

## IMPLICATIONS AND FUTURE DIRECTIONS

There is no doubt that each revision of the classification system provides additional information and improves our understanding of CNS neoplasia, and there are countless positive advances in the 5th edition of the WHO CNS tumor classification (62). In this review, we briefly attempted to describe some of the improvements in major tumor groups (see above) also including some of the controversial areas. We fear that reliance on techniques not available in the overwhelming majority of medical centers of the world suggests that the rift between the global north and the global south is more likely to increase. We have already observed significant applicability problems in our country, even within the referral centers, and increased reliance on “rich” countries to provide guidance. Such concerns have also been raised from other neuropathologists in the global south (63), and despite the genuine response from the leaders of the WHO CNS tumor classification effort (64), there is no satisfactory answer to offer a remedy in everyday practice for pathologists in LMICs. There are significant challenges to the healthcare institutions in LMIC beyond CNS tumor classification adherence, and most pathologists will not be able to utilize the essential and desirable criteria of the new classification for a large number of tumor types and subtypes. Tumor types that can be diagnosed without molecular testing based on WHO CNS essential criteria were displayed inTable 2.

**Table 2:** Tumor types that can be diagnosed without molecular testing based on essential criteria of World Health Organization classification of tumours of the central nervous system 5th edition*.

**Gliomas, Glioneuronal and Neuronal Tumours**
Paediatric-type diffuse low-grade gliomas
Angiocentric glioma
Paediatric-type diffuse high grade gliomas
Diffuse midline glioma, H3 K27-altered
Diffuse hemispheric glioma, H3 G34-mutant
Circumscribed astrocytic gliomas
Pilocytic astrocytoma
Pleomorphic xanthoastrocytoma
Subependymal giant cell astrocytoma
Chordoid glioma
Glioneuronal and neuronal tumours
Ganglioglioma
Gangliocytoma
Rosette forming glioneuronal tumour
Myxoid glioneuronal tumour
Multinodular and vacuolating neuronal tumour
Dysplastic cerebellar gangliocytoma (Lhermitte-Duclos disease)
Central neurocytoma
Cerebellar liponeurocytoma
Posterior fossa ependymoma Group PFA
Myxopapillary ependymoma
Subependymoma
**Choroid Plexus Tumours**
Choroid plexus papilloma
Atypical choroid plexus papilloma
Choroid plexus carcinoma
**Embryonal Tumours**
Medulloblastoma (**)
Medulloblastomas, molecularly defined
Medulloblastoma, WNT-activated
Medulloblastoma, SHH-activated and TP53-wildtype
Medulloblastoma, SHH-activated and TP53-mutant
Medulloblastoma, non-WNT/non-SHH
**Other CNS Embryonal Tumours**
Atypical teratoid/rhabdoid tumour
Cribriform neuroepithelial tumour (provisional entity)
**Pineal Tumours**
Pineocytoma
Pineal parenchymal tumour of intermediate differentiation
Pineoblastoma
Papillary tumour of the pineal region
Desmoplastic myxoid tumour of the pineal region, SMARCB1-mutant
**Cranial and Paraspinal Nerve Tumours**
Schwannoma
Neurofibroma
Perineurioma
Hybrid nerve sheath tumours
Malignant melanotic nerve sheath tumour
Malignant peripheral nerve sheath tumour
Cauda equina neuroendocrine tumour (CNS paraganglioma)
**Meningiomas**
**Mesenchymal, Non-Meningothelial Tumours Involving** **the CNS**
**Soft Tissue Tumours**
Fibroblastic and myofibroblastic tumours
Solitary fibrous tumour
Vascular tumours
Haemangiomas and vascular malformations
Haemangioblastoma
Skeletal muscle tumours
Rhabdomyosarcoma
**Chondro-Osseous Tumours**
Chondrogenic tumours
Mesenchymal chondrosarcoma
Chondrosarcoma
**Notochordal Tumours**
Chordoma
**Melanocytic Tumours**
Diffuse meningeal melanocytic neoplasms
Diffuse meningeal melanocytic neoplasms: Melanocytosis and melanomatosis
Circumscribed meningeal melanocytic neoplasms
Circumscribed meningeal melanocytic neoplasms: Melanocytoma and melanoma
**Haematolymphoid Tumours Involving the CNS**
**Lymphomas**
CNS Lymphomas
Primary diffuse large B-cell lymphoma of the CNS
Immunodeficiency-associated CNS lymphomas
Lymphomatoid granulomatosis
Intravascular large B-cell lymphoma
Miscellaneous rare lymphomas in the CNS
MALT lymphoma of the dura
Other low-grade B-cell lymphomas of the CNS
Anaplastic large cell lymphoma (ALK+/ALK-)
T-cell and NK/T-cell lymphomas
**Histiocytic Tumours**
Erdheim Chester disease
Rosai Dorfman disease
Juvenile xanthogranuloma
Langerhans cell histiocytosis
Histiocytic sarcoma
**Germ Cell Tumours**
**Tumours of the Sellar Region**
Adamantinomatous craniopharyngioma
Papillary craniopharyngioma
Pituicytoma, granular cell tumour of the sellar region and spindle cell oncocytoma
Pituitary adenoma /PitNET
Pituitary blastoma

* Excluding generic NOS types and histologically defined medulloblastoma subtypes ** Although the method for the distinction of the molecular subtypes is not clearly specified in the fifth edition of WHO Classification of CNS tumors, determining the molecular subtype of medulloblastomas is almost always possible with immunohistochemistry. Therefore, they are included in this table

As the “standard” used worldwide, WHO classifications have the responsibility to bring the entire world together under applicable and realistic standards that are at neither the nadir nor the apex of our research endeavors. WHO classifications may resemble constitutional lawmaking in that they may not follow the latest understanding of human condition, but should be well-thought, carefully planned and applicable to most, if not all, circumstances for which the legislation attempts to regulate. Such efforts should follow rigorous protocols (65), considering the three fundamental principles of justice: equality, fairness and accessibility (66). This description may be easily discarded by some who believe that the state-of-the-art and the apex of our research endeavors should guide the classification efforts. However, such an approach fails to recognize the practicability of such “rules and regulations” especially when the entire globe is considered (5,6,8). It is of utmost importance that classifications consist of highly validated and accepted information and should seriously consider their applicability in the real world as well as their reproducibility and pragmatic utility. In a recent study on the challenges of classification systems, Song et al have aptly concluded that “*The arrival of genomic data has dramatically increased the power to peer into the past, but even now, in the midst of the excitement of many new opportunities, it is useful to keep in mind that sometimes the sample series at hand may not be sufficient to support the full ambition of fine-grained classification or to trace the entire evolutionary trajectories*” (67). It is therefore, with great trepidation and concern, we await the application of the new WHO CNS tumors classification scheme across the globe and the advantages and problems that will arise in the “have nots” for which additional solutions need to be identified.

## Conflict of Interest

None.
